# Emerging technologies in wearable sensors

**DOI:** 10.1063/5.0153940

**Published:** 2023-05-31

**Authors:** Francesco Greco, Amay J. Bandodkar, Arianna Menciassi

**Affiliations:** 1 The Biorobotics Institute, Sant'Anna School of Advanced Studies, Viale R. Piaggio 34, 56025 Pontedera, Italy; 2 Department of Excellence in Robotics and AI, Sant'Anna School of Advanced Studies, P.zza Martiri della Libertà, 56127 Pisa, Italy; 3 Institute of Solid State Physics, NAWI Graz, Graz University of Technology, 8010 Graz, Austria; 4 Department of Electrical and Computer Engineering, North Carolina State University, Raleigh, North Carolina 27606, USA; 5 Center for Advanced Self-Powered Systems of Integrated Sensors and Technologies (ASSIST), North Carolina State University, Raleigh, North Carolina 27606, USA

## Abstract

This Editorial highlights some current challenges and emerging solutions in wearable sensors, a maturing field where interdisciplinary crosstalk is of paramount importance. Currently, investigation efforts are aimed at expanding the application scenarios and at translating early developments from basic research to widespread adoption in personal health monitoring for diagnostic and therapeutic purposes. This translation requires addressing several old and new challenges that are summarized in this editorial. The special issue “Emerging technologies in wearable sensors” includes four selected contributions from leading researchers, exploring the topic from different perspectives. The aim is to provide the *APL Bioengineering* readers with a solid and timely overall vision of the field and with some recent examples of wearable sensors, exploring new research avenues.

## INTRODUCTION

The focus of this *Special Issue* in *APL Bioengineering* is to present emerging technologies in wearable sensors, a rapidly growing field of research with strong interdisciplinary crosstalk.^1^ Contributions from materials science and engineering, mechanical/electronic/information engineering and bioengineering, analytical chemistry, and medicine have indeed led to tremendous progress in recent decades. Ever more complex and smarter devices have emerged in this maturing field: wearable sensors' capabilities today span over biophysical and biochemical sensing.^2,3^ Often these devices feature integrated powering and communication capabilities, as needed for a fully operational and autonomous system. Still many challenges remain open with new ones emerging due to new personal monitoring needs in health and sports.

**FIG. 1.**
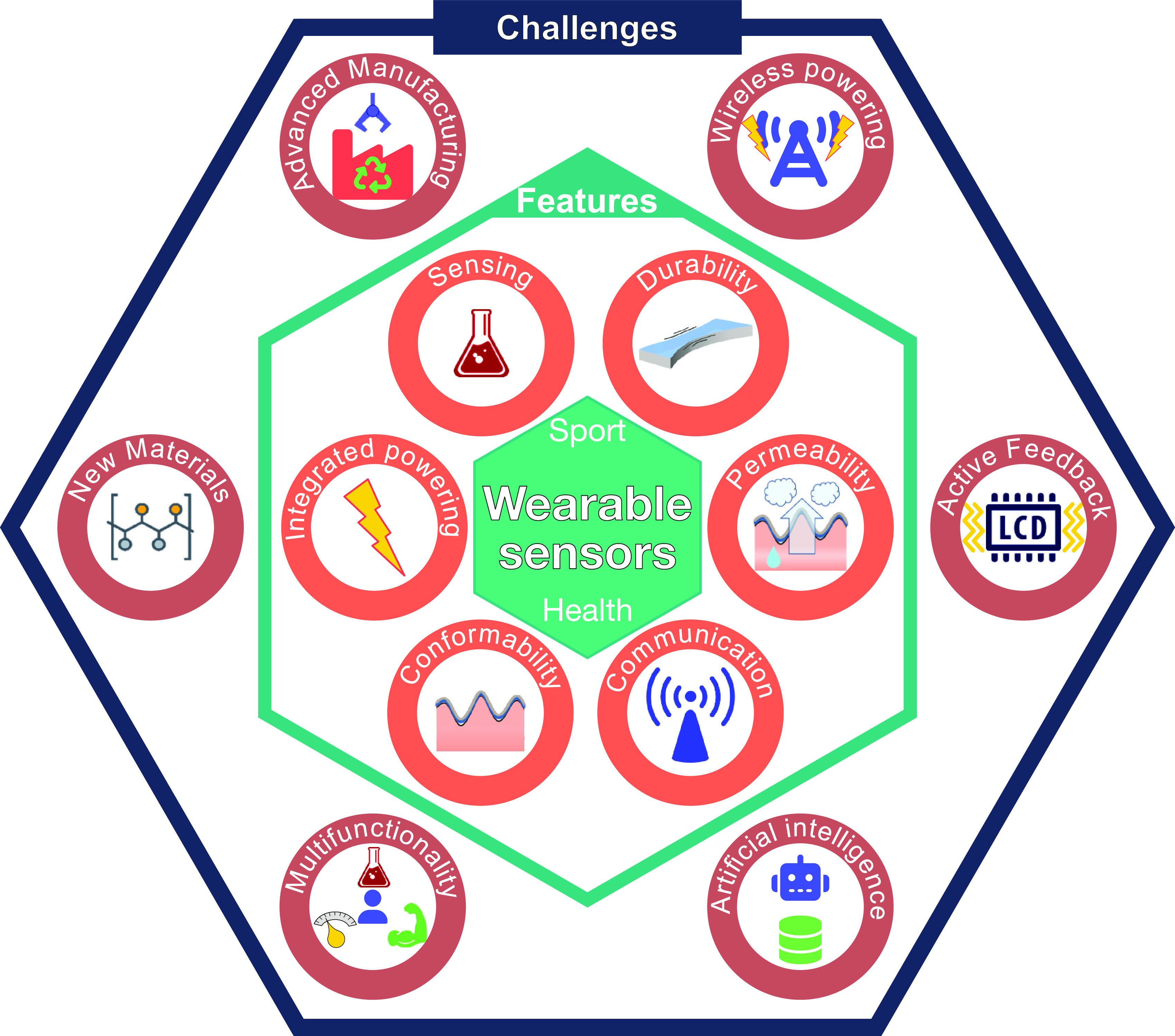
Technological features and challenges of wearable sensors for applications in sport and health. Reprinted with permission from K. Keller (Graz University of Technology, Austria).

Features and challenges of wearable sensors are schematically summarized in Fig. 1.

A large part of research and development efforts is dedicated to enable long-term and continuous operation of wearables: recording of high fidelity biosignals over weeks (or even months) with minimal/no intervention by the user. These autonomous wearables are sought after for diagnostic, therapeutic, or assistive health purposes. This target poses several technological challenges for the hardware, as regards the materials, design, powering, and mechanics of the sensors.

From a materials viewpoint, the main requirements for wearable sensors are related to skin conformability and biocompatibility. The adopted materials should match the soft mechanics of skin to enable a seamless and unobtrusive interfacing with it. This is even more true for long term/chronic health monitoring. To this aim, various strategies have been investigated, from integration of rigid solid-state electronic components and stretchable metal interconnects into soft elastomeric matrices^4^ to development of intrinsically stretchable organic electronics.^5,6^ Current developments deal with enhanced multifunctionality (i.e., multiparametric sensors, integration of sensors with fluidics or with active feedback, displays^6^) and sustainability issues. The latter are faced by investigating novel renewable materials and low-impact processing as well as addressing their recyclability or degradability after use.^7,8^ Bioinspiration is also taken into account for introducing advanced functionalities in wearable sensors, such as self-healing capabilities.^9,10^

Some challenges, not fully solved in earlier research, require the adoption of new strategies. For example, a new importance has been given to breathability or, more in general, to permeability by air, oxygen, moisture, and chemicals (e.g., biomarkers like metabolites contained in sweat or gaseous compounds, like volatile organic compounds, VOCs) through the devices worn on skin. In earlier developments, wearable epidermal devices were often made of impervious barrier materials, made thinner and designed with special geometries to adjust their stiffness and, in turn, their flexibility and conformability to the skin.^4^ However, especially during prolonged use, these materials are far from optimal as they cannot provide the optimal thermal and mass exchange required for comfortable use. This problem is now tackled by several researchers through the adoption and testing of ultrathin transferrable temporary tattoo sensors,^11,12^ novel nanostructured materials, fiber-like and porous matrices, and smart functional textiles. Connected with the challenge of having a seamless, highly stable interfacing with the user skin, a great deal of effort is also made in the topic of smart adhesives. The challenge here is to provide sufficient means of adhesion—which, for example, produces a reduction of motion artifacts in the recorded biosignals—while not damaging the epidermis nor impairing its properties. Also, new functional materials emerged recently whose properties are extremely interesting for their use in wearable devices. This is the case, for example, of novel semiconductors,^13^ piezoelectric,^14^ piezoresistive,^15^ conductive materials, such as laser-induced graphene^16,17^ and MXenes,^18^ among others.

Another frontier of wearables is represented by multifunctionality: wearables should simultaneously monitor many different parameters, through the embedding of several biophysical and biochemical sensing units. This is, for example, the case of patches for monitoring electrophysiology signals (EMG, ECG, and EOG), together with temperature, humidity, or sweat composition. Since the technologies of the various sensors can be very different, the integration of the various functionalities poses challenges both at the level of compatible processing and in terms of crosstalk and interference.

With more and more functionalities to be embedded, combined with the need for wireless data transmission capability and for continuous monitoring, another aspect becomes fundamental: how to power the electronics embedded in wearables? Typically, Li-based batteries are used but, depending on actual power consumption and estimated time of use, their size and weight can be excessive, thus leading to unsuitable form factors. This is why new strategies for intelligent power management, novel battery technologies, wireless power transfer, and energy harvesting strategies are investigated for wearables.^19^

Other challenges emerge when one considers the need of approaches for data analysis and transfer as well as for embedded artificial intelligence. Rapid advancements in AI/ML and big data analytics are playing a key role in developing wearable technologies that offer more accurate results.^20,21^

Last but not least, when looking for scalability, the design of wearable sensors cannot neglect the economic feasibility of manufacturing and assembly processes, a factor that actually hampered further developments and real adoption of some wearable technologies. This is one of the main reasons why printed electronics and additive manufacturing approaches are extensively investigated and adopted for wearable sensors, through several cost-efficient techniques for device prototyping and manufacturing.

## SUMMARY OF AREAS COVERED

Within the Special Topic “Emerging Technologies in wearable devices” in *APL Bioengineering*, the reader can find two review papers along with two research papers.

An excellent introduction and updated state of the art of current challenges and perspectives of wearable sensors is provided in the review by Stuart *et al.*,^22^ where all the aforementioned aspects are discussed in detail. The review summarizes the approaches toward long-term/continuous biosignal monitoring with wearables, highlighting the specific attributes of this class of devices, the barriers, and most promising solutions for expanding the current capabilities. The reader is guided to understand how overall the challenges and efforts in the field have a single common goal: increasing user compliance and adoption of wearable medical devices. This can be affected not only by standard technology performance criteria (i.e., related to the hardware and the sensing ability of the wearable) but also by more complex and subtle considerations related to comfort, usability in a social scenario, and perceived value by the user. Based on a decade or so of data on wearables and their adoption in various scenarios, just recently researchers started considering also these aspects when designing next generation wearables. Also, the review touches the important and timely aspect of how to embed artificial intelligence in next generation devices.

A second review by Das *et al.*^23^ focuses on some material aspects of wearables, and, in particular, on how to achieve the key material attributes of skin conformability, breathability, and biocompatibility through the use of electrospun polymer materials. First, an introduction to fundamentals of electrospinning and electrospun materials is provided. Then, wearable electronic devices and systems that are developed using electrospinning are reviewed, by subdividing them in various classes, depending on applications. The review discusses in detail the use of electrospun polymer fibers in wearables for various subcomponents: biophysical and biochemical sensors (electrophysiological, motion, gas sensors), energy storage (batteries, supercapacitors) or energy harvesting devices (photovoltaics, nanogenerators, rectenna), and transistors. In all these examples, electrospun polymer fibers are combined with organic and inorganic functional materials to achieve the needed functionality.

The two research papers included in this collection explore new strategies for the development of a biophysical^24^ and a biochemical^25^ wearable sensor.

The investigation of novel tailor-made green ink formulations for ink-jet printed wearable sensors is discussed by Galliani *et al.*^24^ Here, a balanced approach is proposed for obtaining high resolution printing, scalable manufacturing, good electrical and mechanical properties, and safe use on skin by tailoring the formulation of a workhorse of printed electronics: the conductive conjugated polymer poly(3,4 ethylene dioxithiophene), PEDOT:PSS. This is achieved with the use of green non-hazardous additives in order to address sustainable manufacturing and biocompatibility. The proposed formulation is studied in detail, and ink-jet printing on various substrate materials is tested. A proof of concept application of a printed wearable touch sensor on a non-woven fabric substrate, capable of tracking human steps, is provided.

An electrochemical sensor based on laser-induced graphene (LIG) capable of analyzing multiple analytes in sweat is discussed by Vivaldi *et al.*^25^ The authors leverage the various attributes of LIG, which include tailorable morphology, specific surface area, wettability, conductivity, and chemical composition, to develop flexible electrodes on thin, flexible polyimide sheets. An unobtrusive paper sweat sampler is interfaced with the LIG-based electrodes for rapid capture of sweat from the wearer's skin. The authors show that the LIG-based electrodes can simultaneously detect uric acid and tyrosine with detection in the micromolar range. Moreover, the authors demonstrated that by functionalizing the electrodes with an indoaniline derivative, they could use the same sensor for detecting sweat pH as well, thus illustrating the sensor's ability to detect multiple biomarkers. Such multi-parametric sensing is especially attractive for wearable applications as it enables reduction of the overall device complexity.

## CONCLUSIONS

Wearable sensors hold great promise in offering unprecedented capabilities in capturing the status of human physiology in real time. This truly interdisciplinary field is based on developments in biomedical engineering, biomechanics, electrical engineering, analytical chemistry, and materials synthesis with increasing contributions from experts in machine learning and artificial intelligence.

In line with the *APL Bioengineering*'s vision, this special issue provides readers with a unique collection of articles that cover different aspects of wearable sensor technologies. In addition, this collection illustrates clearly how researchers with disparate skill sets are working together to solve complex problems with the goal to develop the next generation of wearable sensors that could provide comprehensive insight into a person's well-being.
